# Forskolin, an Adenylcyclase/cAMP/CREB Signaling Activator Restoring Myelin-Associated Oligodendrocyte Destruction in Experimental Ethidium Bromide Model of Multiple Sclerosis

**DOI:** 10.3390/cells11182771

**Published:** 2022-09-06

**Authors:** Tarun Kapoor, Sidharth Mehan, Manisha Suri, Nidhi Sharma, Nitish Kumar, Acharan S. Narula, Abdulrahman Alshammari, Abdullah F. Alasmari, Metab Alharbi, Mohammed A. Assiri, Reni Kalfin

**Affiliations:** 1 Division of Neuroscience, Department of Pharmacology, ISF College of Pharmacy (An Autonomous College), Moga 142001, Punjab, India; 2 Narula Research, LLC, 107 Boulder Bluff, Chapel Hill, NC 27516, USA; 3 Department of Pharmacology and Toxicology, College of Pharmacy, King Saud University, P.O. Box 2455, Riyadh 11451, Saudi Arabia; 4 Institute of Neurobiology, Bulgarian Academy of Sciences, Acad. G. Bonchev St., Block 23, 1113 Sofia, Bulgaria; 5 Department of Healthcare, South-West University “NeofitRilski”, Ivan Mihailov St. 66, 2700 Blagoevgrad, Bulgaria

**Keywords:** adenylcyclase, demyelination, Ethidium Bromide, Forskolin, multiple sclerosis

## Abstract

Multiple sclerosis (MS) is a chronic neurodegenerative disease marked by oligodendrocyte loss, which results in central neuronal demyelination. AC/cAMP/CREB signaling dysregulation is involved in the progression of MS, including mitochondrial dysfunctions, reduction in nerve growth factors, neuronal inflammation, apoptosis, and white matter degeneration. Our previous research has shown that Forskolin (FSK), a naturally occurring direct adenylyl cyclase (AC)/cAMP/CREB activator, has neuroprotective potential to alleviate pathogenic factors linked with numerous neurological abnormalities. The current study intends to explore the neuroprotective potential of FSK at doses of 40 mg/kg and 60 mg/kg alone, as well as in combination with conventional medicines, such as Fingolimod (FNG), Donepezil (DON), Memantine (MEM), and Simvastatin (SIM) in EB-induced demyelinated experimental MS rats. Adult Wistar rats were divided into nine groups, and EB was infused stereotaxically in the rat brain’s intracerebropeduncle (ICP) area. Chronic gliotoxin EB treatment results in demyelination as well as motor and cognitive dysfunctions. FSK, combined with standard medications, improves behavioral dysfunctions, such as neuromuscular and motor deficits and memory and cognitive abnormalities. Following pharmacological treatments improved remyelination by enhancing myelin basic protein and increasing AC, cAMP, and CREB levels in brain homogenates. Furthermore, FSK therapy restored brain mitochondrial-ETC complex enzymes and neurotransmitter levels while decreasing inflammatory cytokines and oxidative stress markers. The Luxol fast blue (LFB) stain results further indicate FSK’s neuroprotective potential in preventing oligodendrocyte death. Therefore, the results of these studies contribute to a better understanding of the possible role that natural phytochemicals FSK could have in preventing motor neuron diseases, such as multiple sclerosis.

## 1. Introduction

Multiple sclerosis is an autoimmune disorder characterized by the destruction of oligodendrocytes and axonal loss causes neuronal inflammatory demyelination in the CNS [1]. Motor and cognitive deficits, fatigue, disorientation, blurred vision, and slurred speech are all the clinical phenotypes that appeared in the early to last stage of MS suffering patients [2]. Globally, approx. 2.8 million people aged 20 to 40 live with MS [3]. It has been reported that women have a twice higher risk than men due to the disease’s persistent and idiopathic nature [4]. In India, approx. 1.33/100,000 persons were diagnosed with MS [5].

Demyelination and axonal loss are caused by neuronal inflammation, which is directly involved in the formation of demyelinating lesions and oligodendrocyte loss in the white and gray matter of the brain [6]. Ethidium Bromide (EB) accumulates within mitochondria and contributes to the inhibition of mitochondrial DNA (mtDNA) replication and translation, which leads to a disruption in mitochondrial functioning [7]. Over the last ten years, our research group has successfully established and validated the EB-induced demyelinated experimental animal model of MS [8,9,10].

Cyclic adenosine monophosphate (cAMP) functions as a secondary messenger, regulating neuronal signaling, synaptic plasticity, and long-term memory by activating cAMP/CREB, brain-derived neurotrophic factors (BDNF), and nerve growth factors (NGF), all of which are associated with the myelination of healthy neurons [11]. BDNF and NGF are neurotrophins produced by neurons, platelets, and oligodendrocytes. Thus, they can play an important role in the progression of MS. BDNF and NGF help to maintain the structural and functional integrity of neurons, neuroprotection, myelination, as well as influence their growth and differentiation through the activation of the CREB and Akt pathways [12]. The production of cAMP from ATP is significantly influenced by adenylcyclase catalysis [13]. cAMP activates protein kinase A (PKA), which phosphorylates numerous target proteins and upregulates the CREB pathway, involved in axonal regeneration and inflammatory responses [14,15]. Degeneration of oligodendrocytes and the inability to repair neurons after immunological stress are the primary processes of demyelination in MS, which is caused by the dysregulation of cAMP and CREB [16].

Forskolin (FSK) is a diterpene derived from the Lamiaceae plant *Coleus forskohlii*, a selective AC activator that activates cAMP by interacting with cyclase and N-protein [17]. FSK promotes neurotransmitters that increase the conversion of ATP into cAMP and intracellular level of cAMP/CREB signaling, both of which are important in the prevention of several neurological dysfunctions, including myotrophic lateral sclerosis (ALS), autism, and Huntington’s diseases (HD) [18,19,20,21].

Several signaling pathways are thought to be involved in the progression of MS, making it difficult to treat. As a result, neurologists often prescribe a combination of drugs to treat MS with several food-derived factors (FDF). People with MS, especially with progressive MS, daily require the prescription of dietary supplements, especially with progressive MS, which requires the prescription of dietary supplements associated with DMT. Several FDF have been associated with a specific biological and anti-inflammatory effect as well as clinical improvement. In this scenario, developing other FDF that would be active against fatigue, mood disorders, and other secondary symptoms in MS is desirable [22]. Conventional drugs used to address symptomatic relief in MS patients include Fingolimod (FNG), Memantine (MEM), Simvastatin (SIM), and Donepezil (DNZ). FNG is the first orally approved drug with high disease modifying efficacy used for the treatment of multiple sclerosis (MS) because it works as a sphingosine-1-phosphate (S1P) modulator [23]. The Akt and ERK pathways, which are directly involved in the phosphorylation of CREB, are activated as a result of FNG [11,24]. It also improves myelination and remyelination, neuron survival, proliferation, differentiation, and migration and reduces apoptosis by increasing OPC [25]. DON is an acetylcholinesterase inhibitor used to treat mild to severe memory impairment and cognitive dysfunction. DON prevents neuronal cell death, promotes neurogenesis, and increases CREB expression in the brain [26]. DON increases CREB1 expression to promote cell transduction and CREB1 phosphorylation, which increases grid protein synthesis and activation of PKA, a key mediator of the second messenger cAMP [27]. MEM, an NMDA (glutamate receptor antagonist), inhibits the transmission of flow through the NMDA receptor and has been shown to protect against neurodegenerative illness [28,29]. HMG-CoA reductase inhibitor SIM has a crucial role in neuroprotection by reducing oxidative stress, enhancing platelet function, activating the Akt and CREB signaling pathways, and decreasing neuronal inflammation [30].

The current therapy medicines for multiple sclerosis are only geared toward reducing the severity of MS-related symptoms. The fact that the activity of these regular treatments only affects one of the neurotransmitters is the primary reason for the limits of this type of treatment. As a result, we must suggest a pharmaceutical intervention for investigating the AC/cAMP/CREB pathway associated with the BDNF and NGF, which is involved in the advancement of MS.

As a result, this work looks into the involvement of AC/cAMP/CREB downregulation in the progression of experimental MS. In Ethidium Bromide-treated MS-like rats, we explored the role of FSK in behavioral and neurochemical preservation. FSK protects MS rats by upregulating AC/cAMP/CREB and restoring mitochondrial ETC functions by lowering demyelination, as evidenced by studies of neurochemical parameters in biological samples, such as brain homogenates. Our findings suggest that FSK, as an AC activator, could be a promising treatment option for people suffering from MS and other neurodegenerative diseases.

## 2. Materials and Methods

### 2.1. Experimental Animals

The Central Animal House of the ISF College of Pharmacy in Moga, Punjab, provided Wistar rats (weighing 250–300 g, aged 4–5 months, both sexes) for the experimental study. The animals were housed in polyacrylic cages with a wire mesh top and soft bedding under normal conditions in a 12-hour light–dark cycle, a temperature of 23.2 °C, and were given full access to food and water. The experimental procedure was authorized by the Institutional Animal Ethics Committee (IAEC) with registration number 816/PO/ReBiBt/S/04/CPCSEA as protocol no. and protocol number was ISFCP/IAEC/CPCSEA/Meeting No.23/2018/Protocol No. 389 and was according to the norms of the Government of India. Before the experimentation, the animal was acclimated to laboratory conditions.

### 2.2. Drugs and Chemicals

EB was bought from Sigma-Aldrich (St. Louis, MO, USA). The FSK was a complimentary sample that came from the BAPEX in India. Ex gratia provided a standard sample of drugs such as Fingolimod (FNG), Simvastatin (SIM), Donepezil (DON), and Memantine (MEM). ELISA kits (MBP, cAMP, CREB, TNF- α, IL-1β) were purchased from Elabscience, India. The other compounds utilized in the study are all of the analytical grades. Before use, all drugs and chemical solutions were freshly prepared. FSK was dissolved in water (with 2% ethanol) and administered orally (p.o.) [17,19]. FNG dissolved in 80% ethanol containing 2 mMNaOH, SIM dissolved in ethanol, MEM dissolved in 0.9% NaCl solution, and DON was dissolved in methanol.

### 2.3. Experimental Grouping of Animals

**Figure 1:**
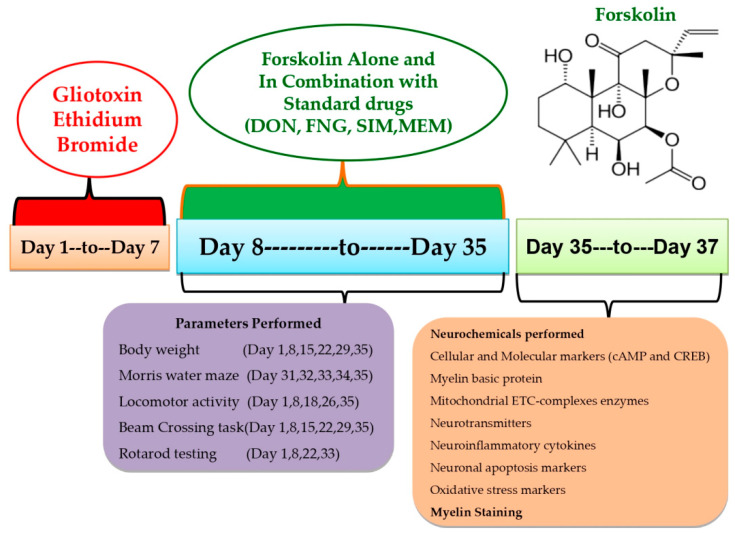
Estimation of behavioral and neurochemical parameters.

This experiment used 54 Wistar rats (4–5 months old, male or female). Rats (3–4 rats per cage) were housed in polyacrylic cages with wire mesh tops and soft bedding (38 cm × 32 cm × 16 cm) at a regulated temperature (22–2 °C) and humidity (65–70%) followed by 12-hour light/dark cycle (lights turned on at 6:00 a.m.). There was no restriction on the animal’s access to food and purified water. Animal bedding was made from sterilized and residue-free wood shavings. Animals of different sex housed separately to run the whole investigation smoothly. Experiments were conducted on animals between 9:00 a.m. and 1:00 p.m. to minimize the effects of circadian rhythms on the results. Researchers carried out an unblinded and well-known experiment. Animals were randomly assigned to the protocol schedule into nine groups. Following experimental groups were employed, such as **Group 1** Sham control (*n* = 6); **Group 2** FSK per se (60 mg/kg p.o.) (*n* = 6); **Group 3** Ethidium Bromide 0.1%/10 μL (EB) (*n* = 6); **Group 4** EB (0.1%/10 μL) + FSK (40 mg/kg p.o.) (*n* = 6); **Group 5** EB (0.1%/10 μL) + FSK (60 mg/kg, p.o.) (*n* = 6); **Group 6** EB (0.1%/10 μL) + FSK (60 mg/kg p.o.) + FNG (0.5 mg/kg i.p.) (*n* = 6); **Group 7** EB (0.1%/10 μL) + FSK (60 mg/kg p.o.) + MEM (5 mg/kg i.p.) (*n* = 6); **Group 8** EB (0.1%/10 μL) + FSK (60 mg/kg p.o.) + DON (1 mg/kg i.p.) (*n* = 6); **Group 9** EB (0.1%/10 μL) + FSK (60 mg/kg p.o.) + SIM (10 mg/kg i.p.) (*n* = 6). The toxin ICP-EB was given from day 1 to day 7 in groups 3 to 9, whereas the protocol drug FSK was given along from day 8 to day 35 in group 4 (40 mg/kg) and group 5 (60 mg/kg), as per the protocol schedule. FSK (60 mg/kg) was administered in combination with standard drug treatment (FNG, DON, MEM, SIM) from day 8 to day 35 in groups 6, 7, 8 and 9, respectively (Figure 1).

### 2.4. Demyelinated Multiple Sclerosis Was Induced in Rats with ICP-EB

From the first to the seventh day of the investigation, chronic EB (0.1 mM/μL/unilateral) infusion was delivered stereotaxically in the ICP region by following the coordinate at AP: −11.6 mm, ML: 2.6 mm, and DV: −7.07 mm to induce MS. This animal model is highly adapted for inducing MS-like symptoms in animals. The laboratory environment was gradually introduced to the rats in order to assimilate them. The surgical procedure began by anesthetizing the rats with ketamine (80 mg/kg). After anesthesia, the animal was placed on stereotaxic equipment. After shaving the scalp hairs, cut about 1–2 cm long from anterior to posterior to expose the scalp to measure and mark the stratum coordinates. Toxin EB was administered through a burr hole into the ICP region of the brain for seven days, according to the protocol schedule using a HalmintonSyring. The cannula was inserted into the hole and fixed using dental cement. Then, the incision was stitched using absorbable suturing thread and a sterile needle. Cannula-implanted rats were housed individually in polyacrylic cages with warm clothes to avoid hypothermia-like conditions in surgical rats and the room temperature was kept at 25 ± 3 °C. The rats were given special attention until they regained spontaneous movement following surgery. After surgery, milk and glucose water were kept in a cage to minimize physical trauma. To avoid sepsis, rats were given gentamycin intraperitoneally (i.p.) for three days. On the sutures, lignocaine gel was applied to relieve pain. The sutures were dusted with Neosporin powder to avoid bacterial infection. FSK, as well as the conventional medications DON, SIM, FNG, and MEM, were given chronically from day 8 to day 35. Tests including the Morris water maze, locomotor activity, and the rotarod were carried out on predetermined days. All animal brains were sacrificed for biochemical, inflammatory, and neurochemical testing on the 36th day of the protocol’s execution schedule [8,10].

### 2.5. Body Weight Measurement

The animal’s weight was measured according to the protocol on the 1st, 8th, 15th, 22nd, 29th, and 35th days of the experimentation. Body weight was measured to see the effect of EB and drugs (FSK, FNG, DON, MEM, and SIM) on food and water intake [31].

### 2.6. Behavioral Parameters

Motor neurons in the CNS were damaged after the administration of EB characterized by destruction of oligodendrocytes, depletion of MBP, demyelination of neurons, and release of cytokines. All these innervations take part in the initiation, management, and coordination of behavioral and motor functions in the brain. In this intervention, we check the level of NTs that support our aforementioned explanation. These are the behavioral parameters that had to be evaluated.

#### 2.6.1. Morris Water Maze (MWM) Task

Memory and cognitive impairment were evaluated by using the MWM test. According to the protocol schedule, the Morris water maze was used to assess the escape latency task (ELT) on the 31st, 32nd, 33rd, and 34th days. ELT is the time (in seconds) the rats took to get to the platform. During the 35th day of the experiment, a hidden platform was added to the Morris water maze apparatus and allowed the rats to swim about for 120 s to record the time spent in the target quadrant of the apparatus (TSTQ). The TSTQ shows that memory consolidation has occurred after teaching [32].

#### 2.6.2. Locomotor Activity (LA)

Researchers used a test to evaluate the motor coordination in the animals that is LA. All animals’ LAs were analyzed on the 1st, 8th, 18th, 26th, and 35th days per the protocol schedule. The LAs of animals were assessed by using an Actophotometer. Each animal was placed on the actophotometer for 5 min. LA was checked as per the number of beams crossed by the animals in 5 min [33].

#### 2.6.3. Beam Crossing Task (BCT)

Gait irregularities and foot slips were measured by using a BCT test. Motor coordination abilities were examined on the 1st, 8th, 15th, 22th, 29th, and 35th days. The number of foot slips that occurred for two minutes in each trial was recorded [34].

#### 2.6.4. Rotarod Test

To examine muscle coordination, a rotarod test was performed. The experiment was conducted on the 1st, 8th, 22nd, and 33rd days. The rotational speed was set to 15 revolutions per minute (rpm). Each rat’s fall-off time (seconds) was recorded for five minutes [9].

### 2.7. Biological Samples Preparation

#### Homogenized Brain Preparation

The decapitation of animals took place on day 36. Isotonic ice-cold saline solution was used for flushing and cleaning the brains. Afterwards, homogenized brain slices were placed in a 0.1 M phosphate buffer with a 7.4 phosphate buffer kept at a cold temperature. After centrifuging the homogeneous material at 10.500× *g* for 15 min, the supernatant was separated. The whole-brain homogenate was used to assess oxidative stress, neurotransmitter, neurochemical, and cellular and molecular changes [35].

### 2.8. Analysis of Cellular Markers

#### Myelin Basic Protein (MBP) Concentration in Brain

Commercial ELISA kits (E-EL-R0010/MBP/Elabsciences, Wuhan Hubei, China) were used to determine the quantity of MBP in the brain. Amounts are presented as g/mg of protein [36].

### 2.9. Analysis of Mitochondrial ETC Complexes Enzyme Concentration in Brain

#### 2.9.1. A Post-Mitochondrial Supernatant (PMS) Prepared from Homogenate of Rat’s Whole Brain

The entire rat brain was homogenized, and the supernatant was used for further research as rat brain PMS after centrifuging at 5000 rpm for 20 min at 4 °C. The unprocessed mitochondrial fraction was created using differential centrifugation. A 1:10 ratio of 0.1 M sodium phosphate buffer (pH 7.4) and the pellet produced during PMS production were processed at 4 °C for 60 min with gentle stirring. The mixture was centrifuged for 30 min at 16,000× *g* and 0 °C. After that, the pellets were resuspended in the same buffer before 250 mmol/L more sucrose were added. Three rounds of centrifugation are necessary to obtain enough of the buffered sucrose solution to isolate the mitochondrial fraction for further analysis [33].

#### 2.9.2. Analysis of Mitochondrial ETC Complex-I (NADPH Dehydrogenase) Protein Concentration in Brain

The complex-I activity was determined by measuring the NADH oxidation rate at 340 nm in an assay medium for three minutes at 37 °C. The rotenone-sensitive activity was found due to complex-I when reactions were carried out with or without the 2 mM rotenone compound. The activity of complex-I was measured in nM/mg protein [37].

#### 2.9.3. Analysis of Mitochondrial ETC Complex-II (Succinate Dehydrogenase/SDH) Protein Concentration in Brain

The absorbance at 490 nm was determined spectrophotometrically (Shimadzu, UV−700) by combining 50 μL of gradient fraction homogenate with 0.3 mL of sodium succinate solution. The chromophore molar extinction coefficient (1.36104 M1 cm1) was used to determine the results, which were then reported as INT-decreased nM/mg protein [19].

#### 2.9.4. Analysis of Mitochondrial ETC Complex-IV (Cytochrome Oxidase) Protein Concentration in Brain

Phosphate buffer, which included 75 millimolar moles of cytochrome-c reductase, was used as the assay solution. The reaction was started using a solubilized mitochondrial sample, and the absorbance change was measured at 550 nm for 2 min. Activity of complex-IV was measured in nM/mg protein [38].

#### 2.9.5. Analysis of Mitochondrial ETC Complex-V (ATP Synthase) Protein Concentration in Brain

Aliquots of homogenates were sonicated in ice-cold perchloric acid (0.1 N) to deactivate ATPase immediately after homogenization. Centrifuged supernatants were neutralized with 1N NaOH and stored at −80 °C until analysis following centrifugation (14,000× *g* at 4 °C for 5 min). A reverse-phase HPLC (Perkin Elmer) was used to determine the ATP concentration in the supernatant. As per the dissolving standard, we created an ATP reference solution at 254 nm for detection [39].

#### 2.9.6. Analysis of Mitochondrial CoQ10 Concentration in Brain

HPLC was used to measure CoQ10 levels in brain tissue and mitochondria. Mitochondria were homogenized in 0.5 mL potassium phosphate buffer (pH 7.4) containing 1 mM dithiothreitol. The homogenate was treated with 2 mL of ethanol with 1 μL/mL butylated hydroxytoluene. Ubiquinone was extracted using 5 mL hexane. Hexane layer dried under nitrogen after agitation with 4 mL of hexane. The residue was diluted in 100 mL of BHT-containing ethanol and analyzed using HPLC. A C18 column with a mobile phase menthol:hexane (90:10 *v/v*) was used to measure CoQ10 at 275 nm, and the results were expressed as nM/g protein [39].

### 2.10. Analysis of Molecular Markers

#### cAMP and CREB Protein Concentration in Brain

Commercial ELISA kits were used to detect the immunoreactivity of homogenized tissue to estimate the level of cAMP and CREB (E-EL-R0056/cAMP; E-EL-R0289/CREB Elabsciences, Wuhan Hubei, China) in rat brain homogenate. The amount was presented as pg/mL [18].

### 2.11. Analysis of Neuroinflammatory Biomarkers

#### TNF- α and IL-1β Protein Concentration in Brain

Kits from E-EL-R0019/TNF-α; E-EL-R0012/IL-1β Elabscienes (Wuhan Hubei, China) were used to detect the amount of TNF-A and IL-1A in rat brain homogenates. TNF-α and IL-1β activity were expressed as pg/mg protein [40,41].

### 2.12. Analysis of Neurotransmitters

#### 2.12.1. Dopamine Concentration in Brain

Dopamine levels were measured in a striatal tissue sample in rat brain homogenates. Dopamine levels were analyzed in the rat brain using an electrochemical detector (ECD) and (HPLC) ECD Waters, Anaheim, CA, USA. The buffering of the mobile phase was prepared with sodium citrate (pH 4.5)–acetonitrile (87:13, volume/volume). The sodium citrate buffer was prepared from citric acid (10 mM), NaH2PO4 (25 mM), EDTA (25 mM), and 1-heptane sulfonic acid (2 mM). The experiment’s electrochemical conditions used a voltage of 0.75 V and a sensitivity range of 5 to 50 nA. A flow rate of 0.8 mL/min was used for the separation. The 20-mL sample was injected manually. The 0.2 M of perchloric acid homogenizing solution was used to homogenize the rat brain samples, which were then centrifuged at 12,000 g for 5 min. The standard way of expressing dopamine activity is ng/mg [42].

#### 2.12.2. Glutamate Concentration in Brain

Glutamate level was quantified by following the derivatizing with o-phthalaldehyde/b-mercaptoethanol (OPA/b-ME), and this analysis was performed by using ECD, HPLC by HPLC-ECD in the USA. The glutamate activity was expressed as ng/mg protein [43].

### 2.13. Analysis of Oxidative Stress Parameters

#### 2.13.1. Protein Estimation

A Coral protein estimate kit (Biuret method) was utilized in order to determine the amount of total protein present in rat brain homogenates using UV/visible spectroscopy at 540 nm [18].

#### 2.13.2. Lactate Dehydrogenase (LDH) Concentration in Brain

LDH levels were determined by spectrophotometric analysis. At the same time, it catalyzed lactate dehydrogenase and lactate oxidation and reduced the level of NAD to NADH. When NAD levels fell, serum LDH activity rose, indicating an increase in absorption. An LDH activity detection kit (Coral Diagnostics, India) was used to detect LDH levels in rat brain homogenates. LDH level was expressed in IU/L [44].

#### 2.13.3. Acetylcholinesterase (AChE) Concentration in Brain

For the assay, the mixture contained the combination of supernatant (0.05 mL) of phosphate buffer (3 mL) and maintained the pH 8, 5,5′-dithiobis-(2-nitrobenzoic acid) (DTNB) (0.10 mL) known as Ellman reagent, and acetylthiocholine iodide (0.10 mL). The shift in 412 nm absorbance was immediately detectable spectrophotometrically. Supernatant enzymatic activity was measured in M/mg protein [45].

#### 2.13.4. Glutathione (GSH) Concentration in Brain

The approach just outlined can be used to assess the brain’s glutathione depletion concentration in the brain. At 4 °C for one hour, supernatant (1 mL) was coldly digested with 4% sulfosalicylic acid (1 mL). The collected samples were centrifuged at 1200 rpm for 15 min. We added 2.7 mL of phosphate buffer (0.1 M, pH 8) and 0.2 mL of DTNB (0.2 mL) to the supernatant (1 mL). A spectrophotometer measured the immediately generated yellow color at 412 nm. The supernatant glutathione level was reported as M/mg protein [34].

#### 2.13.5. Malondialdehyde (MDA) Concentration in Brain

The MDA end product of lipid peroxidation in brain homogeneity was measured using the Wills technique. The amount of MDA was determined by the spectrophotometer at the 532 nm wavelength after the reaction with thiobarbituric acid. MDA concentration was reported as nm/mg protein [45].

#### 2.13.6. Superoxide Dismutase (SOD) Concentration in Brain

As a substrate, the SOD activity was determined using the spectrophotometric method reported for epinephrine auto-oxidation at pH 10.4. To begin the process, 0.02 mL of epinephrine was added to the brain homogenate supernatant (a total of 0.2 mL) before the mixture was combined with 0.8 mL of pH 10.4 50 mM glycine buffer. The absorbance at 480 nm was measured after 5 min of incubation. SOD activity was measured in nM/mg of protein [46].

#### 2.13.7. Nitrite Concentration in Brain

This colorimetric test was performed using Greiss’ reagent (0.1 percent ethylenediamine dihydrochloride, 1 percent sulfanilamide, and 2.5 percent phosphoric acid) to assess nitrite accumulation in the supernatant, which served as an indicator of nitric oxide generation. Supernatant and Greiss reagent were mixed in equal amounts, incubated for 10 min in the dark, and the absorbance of the combination was measured spectrophotometrically at 540 nm at room temperature. A sodium nitrite standard curve can compute the nitrite concentrations in the supernatant and the nitrite concentration represented as µM/mg of protein [47].

### 2.14. Luxsol Fast Blue (LFB) Staining

For LFB staining, rats were sacrificed, and the whole brain was isolated and collected in 0.1 M concentrated solution of PBS and kept aside for 24 h for dehydration. After dehydration, we paraffinized the brain and then dissected into different sections of the thickness of 5 mm. Further, the rat brains were deparaffinized with different concentrations of alcohol along with xylene. After that, the section was stained with LFB and treated with different concentrations of alcohol [48]. The sections were scanned and captured under the MOTICAM-Ba310 image plus 2.0.

### 2.15. Statistical Analysis

Generated data were analyzed with the help of a Two-Way Analysis of Variance (ANOVA) followed by post hoc Bonferroni’s test, and One-Way ANOVA repeated measures followed by post hoc Tukey’s multi-comparison test to examine the differences between various treatment groups. Two-Way ANOVA was used to analyze the body weight and all the behavioral parameters. In contrast, One-Way ANOVA was used to analyze the relative brain–body weight ratio, demyelination volume, biochemical parameters, and TSTQ analysis. *p* < 0.01 was considered statistically significant. Data were found to be normal, and the sample size was calculated by checking the normality distribution via the Kolmogorov–Smirnov test. The statistical analysis was performed using GraphPad Prism software version 5.03 for Windows (GraphPad Software, San Diego, CA, USA). All the statistical results are presented as the mean and standard deviation (SD).

## 3. Results

### 3.1. Protective Role of FSK on Body Weight in Experimental Multiple Sclerosis Rats

**Figure 2:**
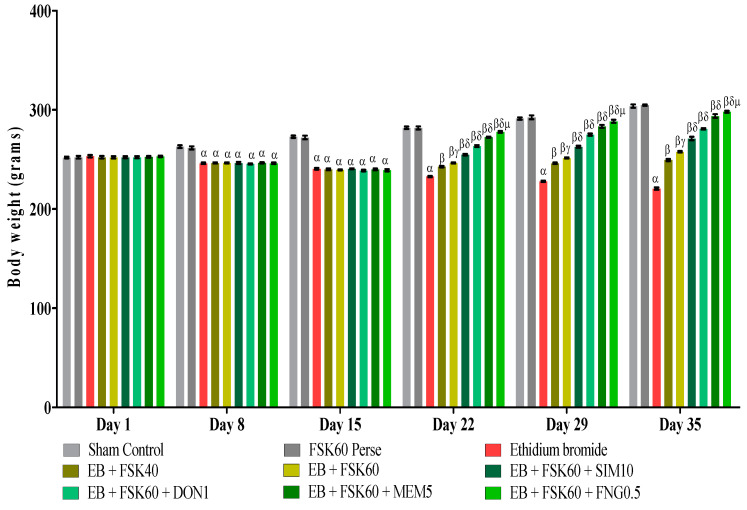
Protective role of FSK on body weight in experimental multiple sclerosis rats. Analysis of Variance (ANOVA) with Bonferroni’s post hoc test was used to evaluate the data, which were presented as mean with SD (*n* = 6). ***^α^*** *EB (p < 0.01) v*/*s Sham control and FSK40 perse; **^β^** EB + FSK40, EB + FSK60, EB + FSK60 + SIM10, EB + FSK60 + DON1, EB + FSK60 + MEM5, EB + FSK60 + FNG0.5 (p < 0.01) v*/*s EB; **^βγ^** EB + FSK60 (p < 0.01) v*/*s EB + FSK40; **^βδ^** EB + FSK60 + SIM10, EB + FSK60 + DON1, EB + FSK60 + MEM5, EB + FSK60 + FNG0.5 (p < 0.01) v*/*s EB + FSK40, EB + FSK60; **^βδμ^*** *EB + FSK60 + FNG0.5 (p < 0.01) v*/*s* *EB + FSK60 + MEM5, EB + FSK60 + DON1, EB + FSK60 + SIM10*.

Body weight was determined on the 1st, 8th, 15th, 22nd, 29th, and 35th days as per the protocol timeline. The ICP-EB (0.1 percent /10 μL)-unilaterally-treated group exhibited a decrease in body weight. Moreover, the chronic administration of FSK40 and FSK60 alone or in combination with conventional medications, such as SIM10, DON1, MEM5, and FNG0.5, considerably improved body weight, as compared to the ICP-EB-treated group in a dose-dependent manner [Two-Way ANOVA:F (40,225) = 830.5, *p* < 0.01]. However, no significant difference was seen in the FSK60-perse-treated group compared to the sham control. Among the selected doses of the drug, the high doses of FSK60 and FNG0.5 were found to be more effective in restoring ICP-EB-induced decreased body weight (Figure 2).

### 3.2. Behavioral Parameters

#### 3.2.1. Protective Role of FSK on Morris Water Maze in Experimental Multiple Sclerosis Rats

##### Improvement in Spatial Learning and Memory

**Figure 3:**
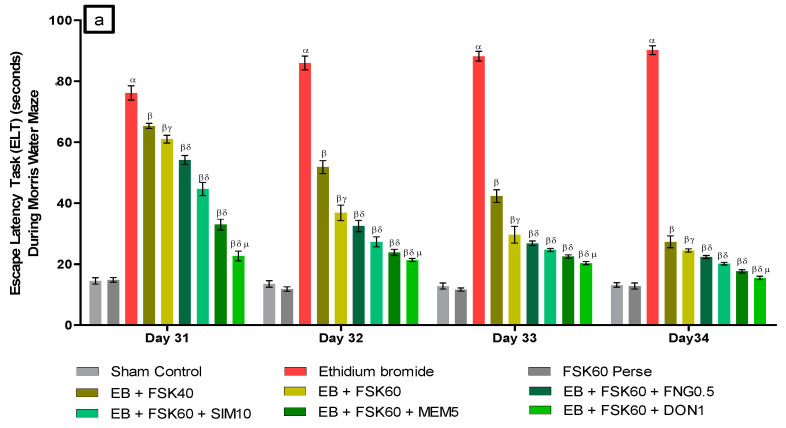

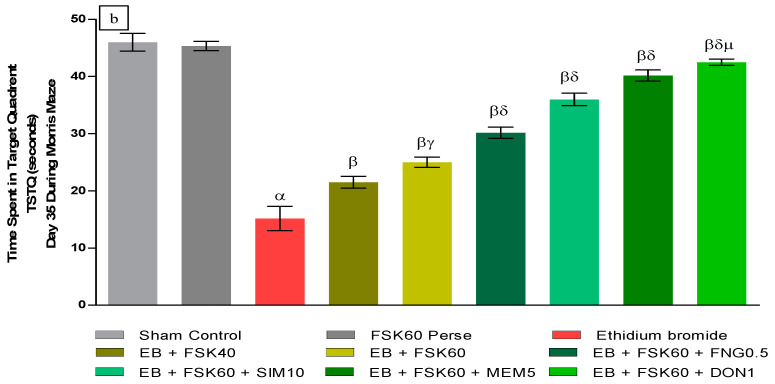
(**a**) Protective role of FSK on escape latency task during Morris water maze in experimental multiple sclerosis rats. (**b**) Protective role of FSK on time spent in targeted quadrant during Morris water maze in experimental multiple sclerosis rats. Analysis of Variance (ANOVA) with Tukey’s test was used to evaluate the data, which were presented as mean with SD (*n* = 6). ***^α^*** *EB (p < 0.01) v*/*s Sham control and FSK40 perse; **^β^** EB + FSK40, EB + FSK60, EB + FSK60 + FNG0.5, EB + FSK60 + SIM10, EB + FSK60 + MEM5, EB + FSK60 + DON1 (p < 0.01) v*/*s EB; **^βγ^** EB + FSK60 (p < 0.01) v*/*s EB + FSK40; **^βδ^** EB + FSK60 + FNG0.5, EB + FSK60 + SIM10, EB + FSK60 + MEM5, EB + FSK60 + DON1 (p < 0.01) v*/*s EB + FSK40, EB + FSK60; **^βδμ^*** *EB + FSK60 + DON1 (p < 0.01) v*/*s* *EB + FSK60 + MEM5, EB + FSK60 + SIM10, EB + FSK60 + FNG0.5*.

*a.*  *Decrease in Escape Latency Task (ELT)*
  ELT was performed on the 31st, 32nd, 33rd, and 34th days as per the protocol schedule of the Morris water maze (MWM). The ICP-EB (0.1 percent /10 μL)-unilaterally-treated group exhibited significantly increased ELT. Moreover, chronic administration of FSK40 and FSK60 alone or in combination with conventional medications, such as FNG0.5, SIM10, MEM5 and DON1, considerably decreased the time in ELT as compared to the ICP-EB-treated group in a dose-dependent manner [Two-Way ANOVA:F (24,135) = 195.0, *p* < 0.01]. However, no significant change was detected in the FSK60 per se treatment group compared to sham control. Among the selected doses of the drug, the high dose of FSK60 and DON1 were found to be more effective in restoring ICP-EB-induced increased ELT (Figure 3a).
*b.*  *Increase in Time Spent in Target Quadrant (TSTQ)*
  TSTQ was performed on day 35 as per the protocol schedule on MWM. The ICP-EB (0.1 percent/10 μL)-unilaterally-treated group had a remarkably decreased TSTQ. Moreover, chronic administration of FSK40 and FSK60 alone or in combination with conventional medications, such as FNG0.5, SIM10, MEM5, and DON1, considerably increased TSTQ as compared to ICP-EB-treated groups in a dose-dependent manner [One-Way ANOVA:F (8,40) = 2.046, *p* < 0.01]. However, no significant change was detected in the FSK60 per se treatment group compared to sham control. Among the selected doses of the drug, the high doses of FSK60 and DON1 were found to be more effective in restoring ICP-EB-induced decreased TSTQ (Figure 3b).

#### 3.2.2. Protective Role of FSK on Locomotor Activity in Experimental Multiple Sclerosis Rats

##### Increase in Locomotion Movement

**Figure 4:**
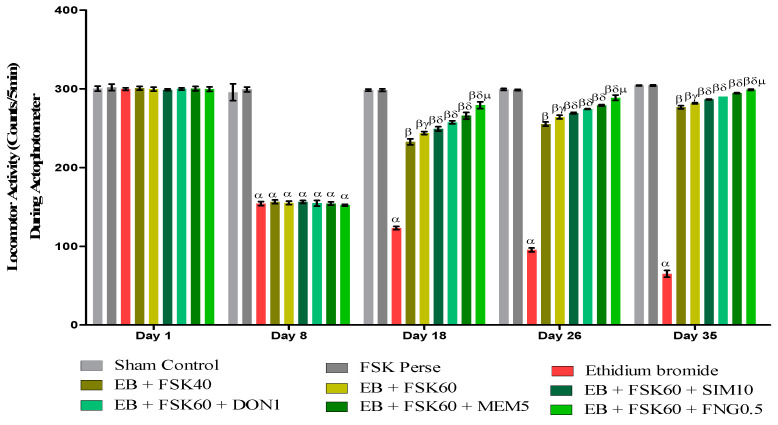
Protective role of FSK during actophotometer in experimental multiple sclerosis rats. Analysis of Variance (ANOVA) with Bonferroni’s post hoc test was used to evaluate the data, which were presented as mean with SD (*n* = 6). ***^α^*** *EB (p < 0.01) v*/*s Sham control and FSK40 perse; **^β^** EB + FSK40, EB + FSK60, EB + FSK60 + SIM10, EB + FSK60 + DON1, EB + FSK60 + MEM5, EB + FSK60 + FNG0.5, (p < 0.01) v*/*s EB; **^βγ^** EB + FSK60 (p < 0.01) v*/*s EB + FSK40; **^βδ^** EB + FSK60 + SIM10, EB + FSK60 + DON1, EB + FSK60 + MEM5, EB + FSK60 + FNG0.5, (p < 0.01) v*/*s EB + FSK40, EB + FSK60; **^βδμ^*** *EB + FSK60 + FNG0.5 (p < 0.01) v*/*s* *EB + FSK60 + MEM5, EB + FSK60 + DON1, EB + FSK60 + SIM10*.

Locomotor activity was performed on the 1st, 8th, 18th, 26th, and 35th days as per the protocol timeline on Actophotometer. The ICP-EB (0.1 percent/10 μL)-unilaterally-treated group exhibited a decreased locomotor activity. Moreover, chronic administration of FSK40 and FSK60 alone or in combination with conventional medications, such as SIM10, DON1, MEM5, and FNG0.5, considerably increased locomotor activity as compared to ICP-EB-treated groups in a dose-dependent manner [Two-Way ANOVA:F (32,180) = 1390, *p* < 0.01]. However, there was no significant change detected in the FSK60 per se treatment group as compared to sham control among the selected doses of the drug; the high doses of FSK60 and FNG0.5 were found to be more effective in the improvement of ICP-EB-induced retardation of locomotor activity (Figure 4).

#### 3.2.3. Protective Role of FSK on Beam Crossing Task in Experimental Multiple Sclerosis Rats

**Figure 5:**
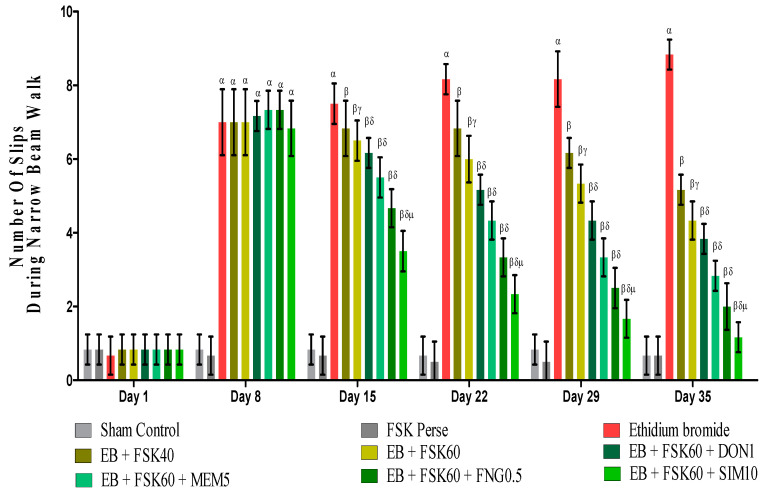
Protective role of FSK during Narrow Beam Walk in experimental multiple sclerosis rats. Analysis of Variance (ANOVA) with Bonferroni’s post hoc test was used to evaluate the data, which were presented as mean with SD (*n* = 6). ***^α^*** *EB (p < 0.01) v*/*s Sham control and FSK40 perse; **^β^** EB + FSK40, EB + FSK60, EB + FSK60 + DON1, EB + FSK60 + MEM5, EB + FSK60 + FNG0.5, EB + FSK60 + SIM10, (p < 0.01) v*/*s EB; **^βγ^** EB + FSK60 (p < 0.01) v*/*s EB + FSK40; **^βδ^** EB + FSK60 + DON1,EB + FSK60 + MEM5, EB + FSK60 + FNG0.5, EB + FSK60 + SIM10, (p < 0.01) v*/*s EB + FSK40, EB + FSK60; **^βδμ^*** *EB + FSK60 + SIM10 (p < 0.01) v*/*s* *EB + FSK60 + FNG0.5, EB + FSK60 + MEM5, EB + FSK60 + DON1*.

BCT was performed on the 1st, 8th, 5th, 22nd, 29th, and 35th days as per the protocol timeline on Narrow Beam Walk (NBW). The ICP-EB (0.1 percent/10 μL)-unilaterally-treated group exhibited dramatically increase in the total number of slips. Moreover, chronic administration of FSK40 and FSK60 alone or in combination with conventional medications, such as DON1, MEM5, FNG0.5, and SIM1, considerably decreased the number of slips as compared to ICP-EB-treated groups in a dose-dependent manner [Two-Way ANOVA:F (40,225) = 40.61, *p* < 0.01]. However, no significant change was detected in the FSK60 per se treatment group compared to sham control. Among the selected doses of the drug, the high doses of FSK60 and FNG0.5 were found to be more effective in reducing ICP-EB-induced increased number of slips in MS rats (Figure 5).

#### 3.2.4. Protective Role of FSK on Grip Strength in Experimental Multiple Sclerosis Rats

**Figure 6:**
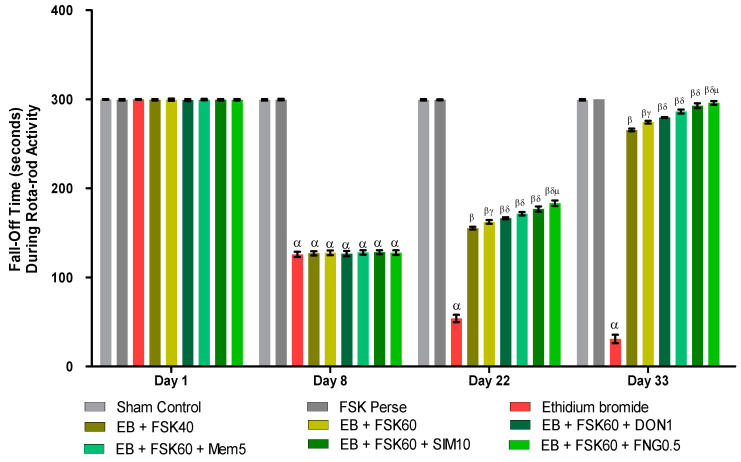
Protective role of FSK rotarod in experimental multiple sclerosis rats. Analysis of Variance (ANOVA) with Bonferroni’s post hoc test was used to evaluate the data, which were presented as mean with SD (*n* = 6). ***^α^*** *EB (p < 0.01) v*/*s Sham control and FSK40 perse; **^β^** EB + FSK40, EB + FSK60, EB + FSK60 + DON1, EB + FSK60 + MEM5, EB + FSK60 + SIM10, EB + FSK60 + FNG0.5 (p < 0.01) v*/*s EB; **^βγ^** EB + FSK60 (p < 0.01) v*/*s EB + FSK40; **^βδ^** EB + FSK60 + DON1,EB + FSK60 + MEM5, EB + FSK60 + SIM10, EB + FSK60 + FNG0.5 (p < 0.01) v*/*s EB + FSK40, EB + FSK60; **^βδμ^*** *EB + FSK60 + FNG0.5 (p < 0.01) v*/*s* *EB + FSK60 + SIM10, EB + FSK60 + MEM5, EB + FSK60 + DON1*.

Rotarod tasks were performed on the 1st, 8th, 22nd, 29th, and 33rd days per the protocol schedule. The ICP-EB (0.1%/10 μL)-unilaterally-treated group showed a random increase in the fall-off time (seconds) on rotarod. Moreover, chronic administration of FSK40 and FSK60 alone or in combination with conventional medications, such as DON1, MEM5, SIM10, and FNG0.5, considerably decreased the fall-off time as compared to ICP-EB-treated group [Two-Way ANOVA:F (24,135) = 3950, *p* < 0.01]. However, no significant change was detected in the FSK60 per se treatment group compared to sham control. Among the selected doses of the drug, the high doses of FSK60 and FNG0.5 were found to be more effective in reducing ICP-EB-induced increased fall of time in MS rats (Figure 6).

### 3.3. Neurochemical Parameters

#### 3.3.1. Protective Role of FSK on the Restoration of Myelination in Experimental Multiple Sclerosis Rats

**Figure 7:**
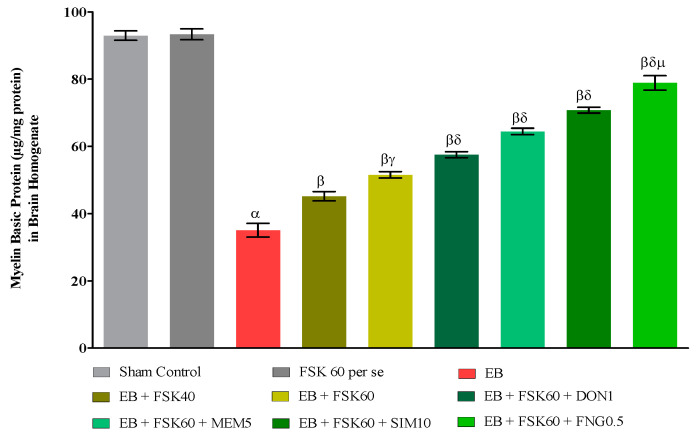
Protective role of FSK on restoration of myelin basic protein in experimental multiple sclerosis rats. Analysis of Variance (ANOVA) with Tukey’s test was used to evaluate the data, which were presented as mean with SD (*n* = 6). ***^α^*** *EB (p < 0.01) v*/*s Sham control and FSK40 perse; **^β^** EB + FSK40, EB + FSK60, EB + FSK60 + DON1, EB + FSK60 + MEM5, EB + FSK60 + SIM10, EB + FSK60 + FNG0.5 (p < 0.01) v*/*s EB; **^βγ^** EB + FSK60 (p < 0.01) v*/*s EB + FSK40; **^βδ^** EB + FSK60 + DON1,EB + FSK60 + MEM5, EB + FSK60 + SIM10, EB + FSK60 + FNG0.5 (p < 0.01) v*/*s EB + FSK40, EB + FSK60; **^βδμ^*** *EB + FSK60 + FNG0.5 (p < 0.01) v*/*s* *EB + FSK60 + SIM10, EB + FSK60 + MEM5, EB + FSK60 + DON1*.

At the end of the treatment, the level of demyelination was evaluated in brain homogenates. The ICP-EB (0.1 percent/10 μL)-unilaterally-treated group exhibited randomly increased demyelination and decreased levels of MBP in brain homogenate. Moreover, chronic administration of FSK40 and FSK60 alone or in combination with conventional medications, such as DON1, MEM5, SIM10, and FNG0.5, considerably decreased demyelination and increased the level of MBP as compared to ICP-EB-treated group [One-Way ANOVA:F (8,40) = 5.617, *p* < 0.01]. However, no significant change was detected in the FSK60 per se treatment group compared to sham control. Among the selected doses of the drug, the high doses of FSK60 and FNG0.5 were found to be more effective in remyelination of ICP-EB-induced demyelination in MS rats (Figure 7).

#### 3.3.2. Protective Role of FSK in Restoring Mitochondrial ETC Complex Enzymes in Experimental Multiple Sclerosis Rats

**Figure 8:**
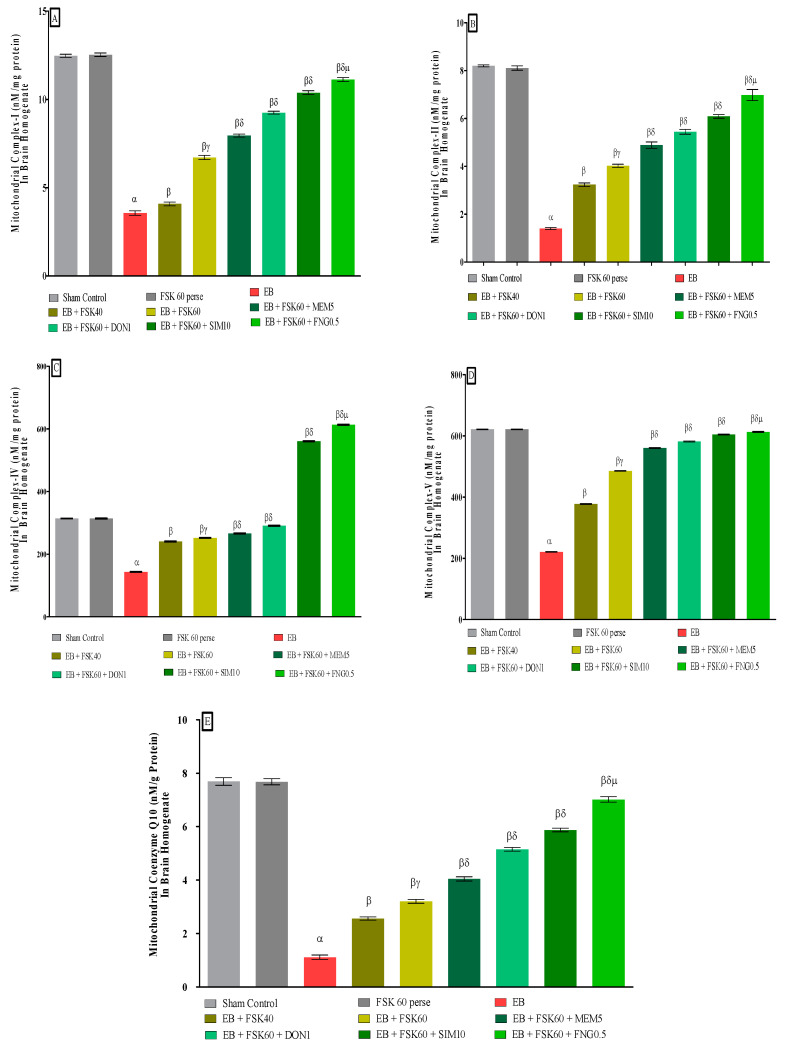
(**A**–**E**): Protective role of FSK in restoring mitochondrial ETC complex enzymes in experimental multiple sclerosis rats. Analysis of Variance (ANOVA) with Tukey’s test was used to evaluate the data, which were presented as mean with SD (*n* = 6). ***^α^*** *EB (p < 0.01) v*/*s Sham control and FSK40 perse; **^β^** EB + FSK40, EB + FSK60, EB + FSK60 + MEM5, EB + FSK60 + DON1, EB + FSK60 + SIM10, EB + FSK60 + FNG0.5 (p < 0.01) v*/*s EB; **^βγ^** EB + FSK60 (p < 0.01) v*/*s EB + FSK40; **^βδ^** EB + FSK60 + MEM5, EB + FSK60 + DON, EB + FSK60 + SIM10, EB + FSK60 + FNG0.5 (p < 0.01) v*/*s EB + FSK40, EB + FSK60; **^βδμ^*** *EB + FSK60 + FNG0.5 (p < 0.01) v*/*s, EB + FSK60 + SIM10, EB + FSK60 + DON1, EB + FSK60 + MEM5*.

At the end of the treatment schedule, activities of mitochondrial complexes were evaluated in brain homogenates. The ICP-EB (0.1 percent/10 μL)-unilaterally-treated group exhibited dramatically decreased activity of mitochondrial ETC complexes (I, II, IV, V, and CoQ10) activity in brain homogenates. Moreover, chronic administration of FSK40 and FSK60 alone or in combination with conventional medications, such as MEM5, DON1, SIM10, and FNG0.5, considerably increased mitochondrial Complex-I [One-Way ANOVA:F (8,40) = 3.970 (*p* < 0.01)] (Figure 8A), Complex-II [One-Way ANOVA:F (245.9,40) = 1.208 (*p* < 0.01)] (Figure 8B), Complex-IV [One-Way ANOVA:F (8,40) = 2.082 (*p* < 0.01)] (Figure 8C), Complex-V [One-Way ANOVA:F (8,40) = 5.057 (*p* < 0.01)] (Figure 8D), and CoQ10 [One-Way ANOVA:F (8,40) = 0.6141 (*p* < 0.01)] (Figure 8E) activity as compared to the ICP-EB-treated group. However, no significant change was detected in the FSK60 per se treatment group compared to sham control. Among the selected doses of the drug, the high doses of FSK60 and FNG0.5 were found to be more effective in restoration and increased the mitochondrial ETC complexes enzymatic activity of ICP-EB induced in MS rats.

#### 3.3.3. Protective Role of FSK in Upregulation of cAMP and CREB Levels in Experimental Multiple Sclerosis Rats

**Figure 9:**
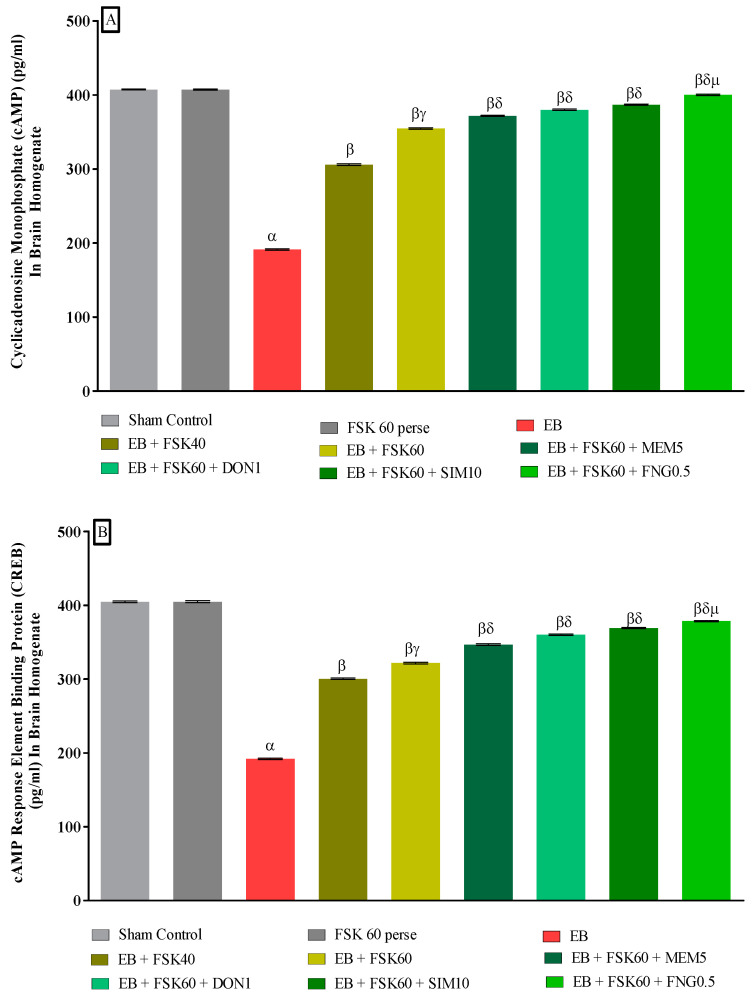
(**A**,**B**): Protective role of FSK in upregulation of cAMP and CREB levels in experimental multiple sclerosis rats. Analysis of Variance (ANOVA) with Tukey’s test was used to evaluate the data, which were presented as mean with SD (*n* = 6). ***^α^*** *EB (p < 0.01) v*/*s Sham control and FSK40 perse; **^β^** EB + FSK40, EB + FSK60, EB + FSK60 + MEM5, EB + FSK60 + DON1, EB + FSK60 + SIM10, EB + FSK60 + FNG0.5 (p < 0.01) v*/*s EB; **^βγ^** EB + FSK60 (p < 0.01) v*/*s EB + FSK40; **^βδ^** EB + FSK60 + MEM5, EB + FSK60 + DON1, EB + FSK60 + SIM10, EB + FSK60 + FNG0.5 (p < 0.01) v*/*s EB + FSK40, EB + FSK60; **^βδμ^*** *EB + FSK60 + FNG0.5 (p < 0.01) v*/*s* *EB + FSK60 + SIM10, EB + FSK60 + DON1, EB + FSK60 + MEM5*.

Molecular markers such as cAMP and CREB levels were estimated in rat brain homogenates at the end of the protocol schedule. The ICP-EB (0.1 percent/10 μL)-unilaterally-treated group exhibited a decrease in the level of cAMP and CREB in brain homogenate. Moreover, chronic administration of FSK40 and FSK60 alone or in combination with conventional medications, such as MEM5, DON1, SIM10, and FNG0.5, considerably improved the level of cAMP levels [One-Way ANOVA:F (8,40) = 0.614 (*p* < 0.01)] (Figure 9A) and CREB levels [One-Way ANOVA:F (8,40) = 0.9545 (*p* < 0.01)] (Figure 9B) activity as compared to the ICP-EB-treated group. However, no significant change was detected in the FSK60 per se treatment group compared to sham control. Among the selected dosages of the drug, the high doses of FSK60 and FNG0.5 were found to be more effective in restoring dopamine and glutamate levels in ICP-EB-induced MS rats.

#### 3.3.4. Protective Role of FSK on Amelioration of Inflammatory Cytokines Levels in Experimental Multiple Sclerosis Rats

**Figure 10:**
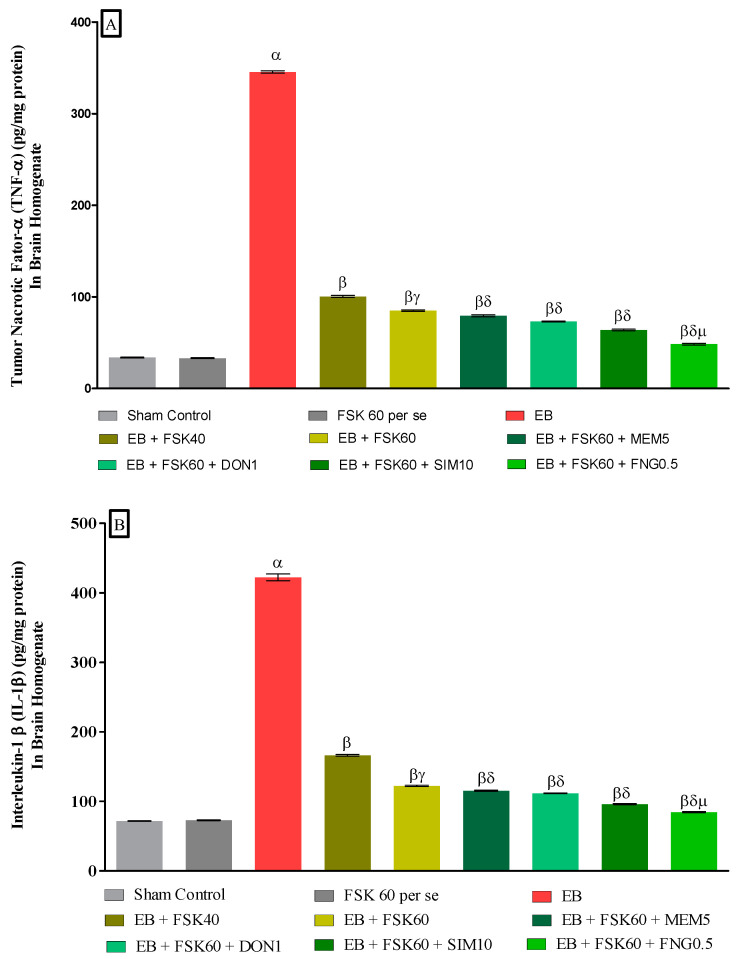
(**A**,**B**): Protective role of FSK on amelioration of inflammatory cytokines levels in experimental multiple sclerosis rats. Analysis of Variance (ANOVA) with Tukey’s test was used to evaluate the data, which were presented as mean with SD (*n* = 6). ***^α^*** *EB (p < 0.01) v*/*s Sham control and FSK40 perse; **^β^** EB + FSK40, EB + FSK60, EB + FSK60 + MEM5, EB + FSK60 + DON1, EB + FSK60 + SIM10, EB + FSK60 + FNG0.5 (p < 0.01) v*/*s EB; **^βγ^** EB + FSK60 (p < 0.01) v*/*s EB + FSK40; **^βδ^** EB + FSK60 + MEM5, EB + FSK60 + DON1, EB + FSK60 + SIM10, EB + FSK60 + FNG0.5 (p < 0.01) v*/*s EB + FSK40, EB + FSK60; **^βδμ^*** *EB + FSK60 + FNG0.5 (p < 0.01) v*/*s* *EB + FSK60 + SIM10, EB + FSK60 + DON1, EB + FSK60 + MEM5*.

At the end of the treatment, neuroinflammatory cytokines such as TNF-α and IL-1β levels were estimated in brain homogenates. The ICP-EB (0.1 percent/10 μL)-unilaterally-treated group significantly increased the level of TNF-α and IL-1β in brain homogenates. Moreover, chronic administration of FSK40 and FSK60 alone or in combination with conventional medications, such as MEM5, DON1, SIM10, and FNG0.5, considerably decreased the level of TNF-α [One-Way ANOVA:F (8,40) = 1.454 (*p* < 0.01)] (Figure 10A) and IL-1β [One-Way ANOVA:F (8,40) = 1.794 (*p* < 0.01)] (Figure 10B) as compared to the ICP-EB-treated group. However, no significant change was detected in the FSK60 per se treatment group compared to sham control. Among the selected doses of the drug, the high doses of FSK60 and FNG0.5 were found to be more effective in the reduction in neuroinflammatory cytokines levels in ICP-EB-induced MS rats.

#### 3.3.5. Protective Role of FSK on Ameliorating Neurotransmitter Alteration Levels in Experimental Multiple Sclerosis Rats

**Figure 11:**
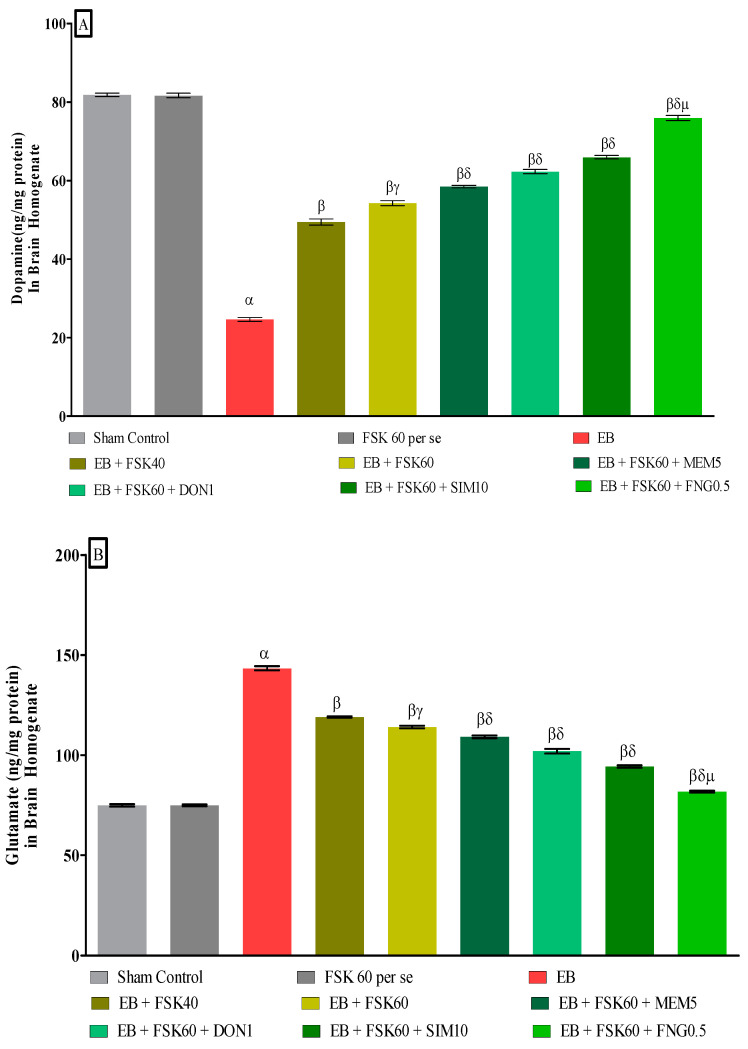
(**A**,**B**): Protective role of FSK on amelioration of neurotransmitter alteration levels in experimental multiple sclerosis rats. Analysis of Variance (ANOVA) with Tukey’s test was used to evaluate the data, which were presented as mean with SD (*n* = 6). ***^α^*** *EB (p < 0.01) v*/*s Sham control and FSK40 perse; **^β^** EB + FSK40, EB + FSK60, EB + FSK60 + MEM5, EB + FSK60 + DON1, EB + FSK60 + SIM10, EB + FSK60 + FNG0.5 (p < 0.01) v*/*s EB; **^βγ^** EB + FSK60 (p < 0.01) v*/*s EB + FSK40; **^βδ^** EB + FSK60 + MEM5, EB + FSK60 + DON1, EB + FSK60 + SIM10, EB + FSK60 + FNG0.5 (p < 0.01) v*/*s EB + FSK40, EB + FSK60; **^βδμ^*** *EB + FSK60 + FNG0.5 (p < 0.01) v*/*s* *EB + FSK60 + SIM10, EB + FSK60 + DON1, EB + FSK60 + MEM5*.

At the end of the treatment, neurotransmitter levels such as dopamine and glutamate were measured in the brain homogenate. The ICP-EB (0.1 percent/10 μL)-unilaterally-treated group showed decreased dopamine and increased glutamate concentrations in the brain homogenates. Moreover, chronic administration of FSK40 and FSK60 alone or in combination with conventional medications, such as MEM5, DON1, SIM10, and FNG0.5, considerably increased the dopamine level [One-Way ANOVA:F (8,40) = 1.432 (*p* < 0.01)] (Figure 11A) and decreased the glutamate level [One-Way ANOVA:F (8,40) = 1.481 (*p* < 0.01)] (Figure 11B) activities as compared to ICP-EB-treated group. However, no significant change was detected in the FSK60 per se treatment group compared to sham control. Among the selected dosages of the drug, the high doses of FSK60 and FNG0.5 were found to be more effective in restoring dopamine and glutamate levels in ICP-EB-induced MS rats.

#### 3.3.6. Protective Role of FSK on Reduction in Oxidative Stress Levels in Experimental Multiple Sclerosis Rats

**Figure 12:**
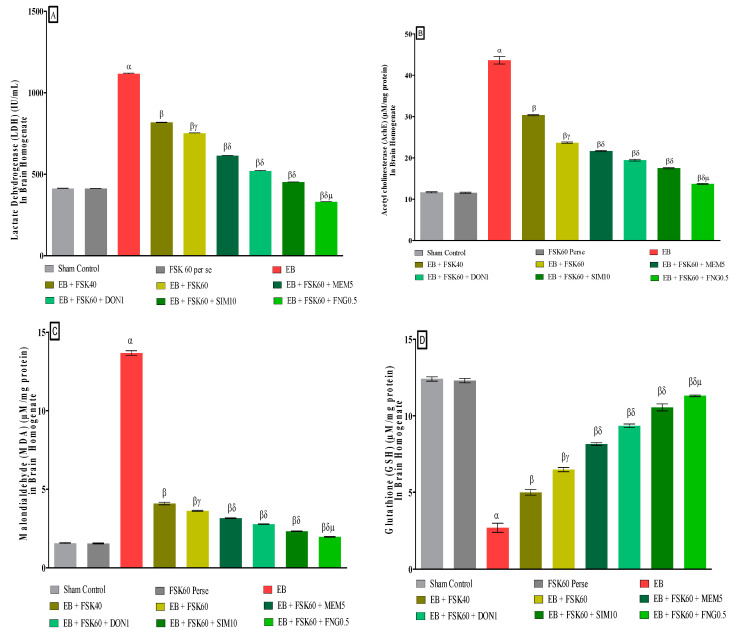

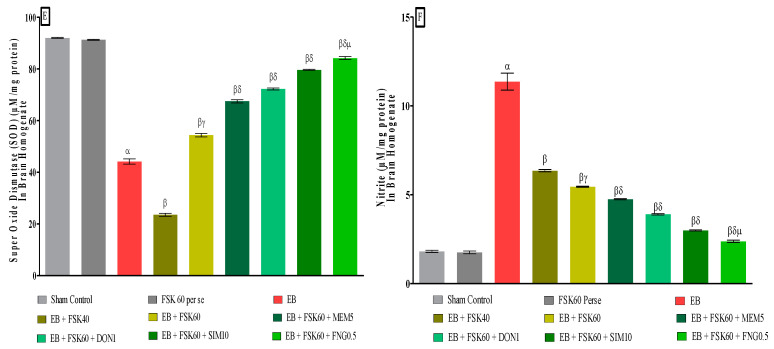
(**A**–**F**): Protective role of FSK on reduction in oxidative stress levels in experimental multiple sclerosis rats. Analysis of Variance (ANOVA) with Tukey’s test was used to evaluate the data, which were presented as mean with SD (*n* = 6). ***^α^*** *EB (p < 0.01) v*/*s Sham control and FSK40 perse; **^β^** EB + FSK40, EB + FSK60, EB + FSK60 + MEM5, EB + FSK60 + DON1, EB + FSK60 + SIM10, EB + FSK60 + FNG0.5 (p < 0.01) v*/*s EB; **^βγ^** EB + FSK60 (p < 0.01) v*/*s EB + FSK40; **^βδ^** EB + FSK60 + MEM5, EB + FSK60 + DON1, EB + FSK60 + SIM10, EB + FSK60 + FNG0.5 (p < 0.01) v*/*s EB + FSK40, EB + FSK60; **^βδμ^*** *EB + FSK60 + FNG0.5 (p < 0.01) v*/*s, EB + FSK60 + SIM10, EB + FSK60 + DON1, EB + FSK60 + MEM5*.

At the end of the treatment, oxidative indicators such as MDA, GSH, nitrite, SOD, AchE, and LDH were measured in the brain homogenate. T**he** ICP-EB (0.1 percent/10 μL)-unilaterally-treated group exhibited dramatically elevated MDA, nitrite, LDH, and AchE levels and decreased the levels of GSH and SOD in the brain homogenates. Moreover, chronic administration of FSK40 and FSK60 alone or in combination with conventional medications, such as MEM5, DON1, SIM10, and FNG0.5 considerably decreased the concentration of MDA [One-Way ANOVA:F (8,40) = 0.4429 (*p* < 0.01)] (Figure 12C), nitrite [One-Way ANOVA:F (8,40) = 1.177 (*p* < 0.01)] (Figure 12E), LDH [One-Way ANOVA:F (8,40) = 1.157 (*p* < 0.01)] (Figure 12A), and AchE [One-Way ANOVA:F (8,40) = 1.932 (*p* < 0.01)] (Figure 12B), and increased the levels of GSH [One-Way ANOVA:F (8,40) = 1.721 (*p* < 0.01)] (Figure 12D) and SOD [One-Way ANOVA:F (8,40) = 0.9711 (*p* < 0.01)] (Figure 12E) as compared to the ICP-EB-treated group. However, there was no significant change detected in the FSK60 per se treatment group as compared to sham control. Among the selected dosages of the drug, the high doses of FSK60 and FNG0.5 were found to be more effective in reducing and regulating oxidative indicators in ICP-EB-caused MS rats.

#### 3.3.7. Protective Role of FSK on LFB Stain in Experimental Multiple Sclerosis Rats

**Figure 13:**
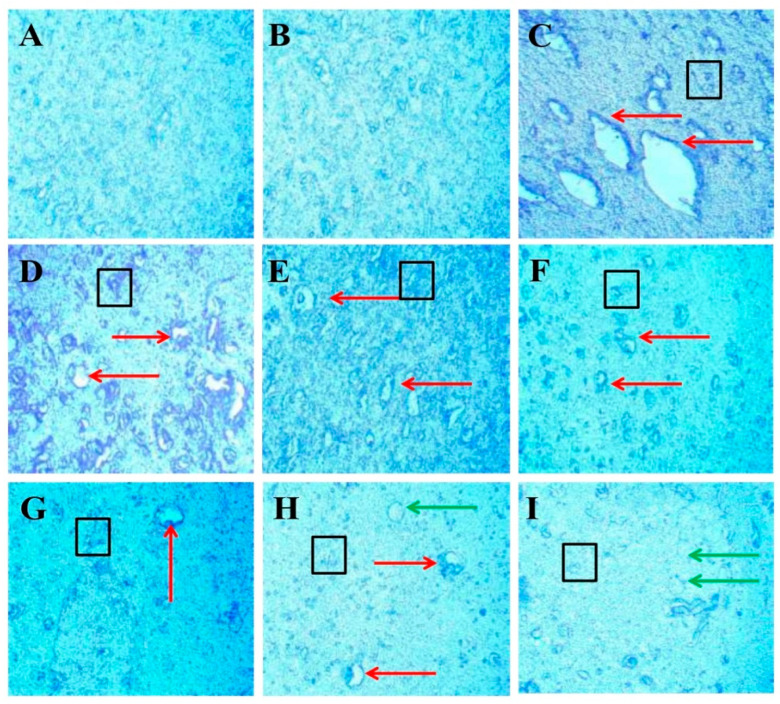
Protective role of FSK on remyelination in experimental multiple sclerosis rats. Brain sections stained with the LFB indicate the demyelinated and remyelinated areas. Clear white lesions show the brain’s demyelination, while blue indicates the normal myelination area in the brain. The red arrow indicates the demyelinated regions caused by the destruction of oligodendrocytes in the brain, the black boxes indicate the swelling of the axons, and the green arrow indicates the shadow of plaques that indicate the remyelination occurred. (**A**) Diagram shows the positive stain intensity with high no. of oligodendrocytes in the normal group. (**B**) Diagram shows no change in the number of oligodendrocytes, and no sign of demyelination occurs when comparing FSK40 per se with the normal group. (**C**) Diagram shows the demyelinating lesions in the brain with axonal swelling in EB-induced rats. (**D**,**E**) Diagrams show the effect of a low dose of FSK40 and a high dose of FSK60 on the reduction in demyelination and axonal swelling. (**F**–**I**) The diagrams showed the significant demyelination and axonal swelling reduction when EB-induced rats were treated with the high dose of FSK60 and standard drugs (F- DON, G-MEM, H-SIM, G-FNG), respectively. (**F**) Diagram also shows remyelination occurs in the form of the shadow of plaques.

Luxsol Fast Blue (LFB) staining shows the content of myelin in the brain section of rats after EB-induced demyelination. The LFB stain helps to determine the demyelination process accelerating in the rat brain after ICB-EB-induced demyelination. To evaluate the effect of FSK alone and with standard drugs on the restoration of demyelination, nine groups compare with each other. LFB analysis provides a quantitative scanned and captured result that significantly increases the demyelination area in the EB-induced rat (Figure 13C). At the same time, treatment groups significantly improve and restore the demyelinating area in the rat brain (Figure 13D–I).

## 4. Discussion

In this work, we evaluate the neuroprotective impact of FSK as an activator of the AC/cAMP/CREP pathways in rats with EB-induced MS. Gliotoxin EB (3,8-Diamino-5-ethyl-6-phenyl-phenanthridinium) is an intercalating dye that induces demyelination in rat brains, a characteristic of MS [49]. EB is responsible for cytotoxic effects due to its binding with DNA strands. The neuronal degeneration associated with motor and sensory deficits can be assessed by directly injecting EB into an ICP region of the brain [50]. EB may trigger microglial inflammation, destroying oligodendrocytes and demyelinating the brain. EB accumulation in mitochondrial cells may cause mtDNA translocation, affecting transcription and replication and lowering oxygen consumption, leading to mitochondrial malfunction and neuronal death. We confirmed an EB-induced animal model of MS employing neurochemical, behavioral, metabolic, and cellular changes in adult rats [8,9,10]. EB was used to induce long-term demyelination in the rat brain’s ICP region for seven days.

FSK is a diterpene produced from the *Coleus forskohlii*, a member of the Lamiaceae family. In a prior investigation, we found mitochondrial dysfunction due to decreased cAMP levels in EB-induced rats [9]. FSK was discovered to be neuroprotective by reversing behavioral, neurochemical, and demyelination changes. The activation of the cAMP and CREB pathways was proven to be a direct activator of AC [38]. FSK has been shown to be a neuroprotective and memory-enhancing property [18]. In the current investigation, we observed that FSK could prevent EB-induced demyelination and improve body weight, memory, and cognition impairments, locomotion, and muscular incoordination [38]. Furthermore, by activating the AC and CREB pathways, FSK was able to restore MBP, cAMP, and CREB while modulating inflammatory markers, neurotransmitters, and oxidative markers in brain homogenate samples [20].

Neurologists prescribe a combination of medications for MS because numerous signaling pathways are implicated in its pathogenesis. Our study uses a combination of drugs (FNG, DON, MEM, and SIM) used in the MS condition. It directly shows correlation with phosphorylation of CREB [11,24]. FNG plays a part in preventing demyelination by increasing the number of oligodendrocytes cells [25]. DON is an acetylcholinesterase inhibitor used to treat memory impairment and cognitive dysfunction by increasing CREB expression in the brain [26]. MEM is an NMDAR antagonist and takes part in neuroregeneration by inhibiting the flow of NMDAR [28,29]. SIM is an HMG-CoA reductase inhibitor that reduces oxidative stress and inflammatory cytokine levels by activating the Akt and CREB pathways [30].

The clinical evidence implies that MS is related to a range of neurological signs and symptoms; as a result, we applied a combination of treatments to slow down the progression of symptoms in the hope that this may help.

Evidence indicates that people with MS tend to lose body weight because they have trouble swallowing food, tiredness, tremors, and postural instability [51]. Throughout this experiment, we observed a decrease in body weight in the EB-treated group [30]. FSK60, on the other hand, was found to be more effective in regaining body weight, both alone and in conjunction with existing treatments.

Memory and cognitive impairments are widespread in MS patients due to the downregulation of cAMP and CREB signaling, which are directly related to glutamate and NMDAR activation [52]. FSK60’s ability to prevent memory deficits in EB-induced memory impairments was one of our study’s most promising findings during the water maze task.

The clinical data suggested that MS patients may experience a motor delay due to the degeneration of striatal neurons [53]. It has been demonstrated that EB can cause motor impairments in experimental animals [33]. FSK60 alone or in combination with conventional drugs protects against motor dysfunction and increases locomotor movement frequency. Muscle weakness and stiffness are frequent MS symptoms that make it difficult for patients to walk normally and maintain their balance, increasing the risk of falling [54].

This study tested rats’ motor coordination and muscular weakness by having them walk on the NBW and grip a rotating rod. FSK60 enhanced the EB-induced rats’ walking and gripping ability in rotarod and beam crossing studies.

This study examines the neurotoxic effects of EB on demyelination, cellular, and molecular markers, mitochondrial ETC complex enzymes, neurotransmitter levels, and inflammatory cytokines.

According to Greene and her colleagues’ clinical studies, the amount of MBP in MS patients decreased by approximately one-third. As a result, MBP is currently being examined as a potential diagnostic for MS [55]. Previous research has demonstrated that a decrease in MBP levels in the EB contributes to the development of MS in rats [36]. In this study, we found that EB-induced demyelination lowers the level of MBP in the rat brain. The combination of FSK60 and FNG0.5 was more successful than the other standard drugs in improving and restoring MBP levels.

Several investigations have shown that mitochondrial malfunction can result in the inadequate activation of ETC complexes. These cause abnormalities in mitochondrial transport, resulting in an imbalance in energy levels and a direct involvement in disease progression [56]. In this work, we determined that EB reduces mitochondrial ETC complex activity (complex-I, -II, -IV, -V, and CoQ10) in the rat brain. Furthermore, long-term administration of FSK60 in combination with FNG0.5 results in significant restoration of mitochondrial ETC complex enzyme levels such as complex-I, -II, -IV, -V, and CoQ10.

Our prior research indicates that EB-induced downregulation of the AC/cAMP/CREB pathway contributes to MS pathogenesis [18]. In contrast, this study found that FSK60 demonstrated a stronger influence on the cAMP/CREB pathway when combined with FNG0.5 than other conventional treatments.

TNF-α and IL-1β are exceptionally rare in the brain tissue of normal individuals. However, larger levels of TNF-α and IL-1β are detected in autopsy studies of active demyelinated lesion areas in MS patients’ brains [57,58]. Furthermore, continuous infusion of Gliotoxin EB has been linked to increased inflammatory cytokines, such as TNF-α and IL-1β [40,41]. The treatment with FSK60 effectively reduces the levels of inflammatory cytokines (TNF-α and IL-1β) in the rat brain.

Researchers observed that an injection of EB was linked to increased levels of dopamine and decreased levels of glutamate [42,43]. Simultaneously, FSK60 combined with MEM5 significantly reduced neurotransmitter glutamate while raising dopamine levels in EB-treated rats.

Oxidative indicators such as AchEs, LDH, MDA, GSH, SOD, and nitrite are widely known. Long-term EB-induced multiple sclerosis in rats was associated with increased oxidative markers (AchEs, LOD, MDA, and nitrite) and a reduction in antioxidants (GSH and SOD) [40]. Moreover, this study showed that EB injection in rat brains altered the level of oxidative markers followed by antioxidants in the pons, hypothalamus, and cerebellum, whereas, FSK60 alone and combined with standard medication considerably improved and restored the level of oxidative markers in the above-stated area.

Demyelinated lesions can arise in MS patients due to a destructive zone of oligodendrocyte cells [48]. LFB staining was used to assess the degree of demyelination in the midbrain [59]. This research reveals that EB-induced MS causes axonal swelling, oligodendrocyte death, and myelin sheath degradation. FSK60, in conjunction with FNG0.5, produces remyelination in the form of plaque shadows and greatly improves demyelinating regions while decreasing axonal swelling in the rat brain.

## 5. Limitations

Based on these findings, we suggested that FSK60 could be used to treat MS-like symptoms. The mechanistic method, on the other hand, must be validated through studies of adenylcyclase genes using knock-in and knock-out techniques. These findings are likely to be confirmed by cellular marker tests, such as Western blot and immunohistochemistry.

According to our survey, FDF would be more beneficial when combined with FSK and could be used in MS therapy as a supplemental treatment. We also recommended further research to determine the relationship and utility of FDF when combined with other MS treatments and DMTs.

## 6. Conclusions

Throughout the research, we concluded that EB-induced demyelination could be prevented by using FSK either by itself or in combination with other standard drugs, i.e., FNG, DON, MEM, and SIM, by activating the AC/cAMP/CREB signaling pathway. According to LFB analysis, we believe that FSK treatment reduced the loss of oligodendrocytes cells in the demyelinated region, which is consistent with the restoration of the myelin basic protein (MBP). This study reveals the effectiveness of FSK in the restoration of the mitochondrial complex as well as the levels of cAMP and CREB. These research findings show the improvement in the level of NTs and inflammatory markers. Our histology findings imply that FSK can prevent neurodegeneration and restore the EB-induced decrease in antioxidant levels. Consequently, this research strongly suggests that FSK could be a promising therapeutic agent for treating MS pathogenic symptoms. This study also highlights the significance of investigating the role of the AC/cAMP/CREB signaling pathway in the pathogenesis of MS, as it could be used as a futuristic approach in the prevention of cellular and molecular dysfunction associated with MS.

## Data Availability

All data generated or analyzed during this study are included in this article. There are no separate or additional files.

## Abbreviations

AC	Adenylylcyclase
Ach	Acetyl choline
Ach	Acetyl Cholinesterase
ALS	Amyotrophic Lateral Sclerosis
ANOVA	Analysis of Variance
ATP	Adenosine Triphosphate
BCT	Beam Crossing Task
BDNF	Brain-Derived Neurotrophic Factor
BHT	Butylated Hydroxytoluene
b-ME	b-Mercaptoethanol
cAMP	Cyclic Adenosine Monophosphate
CoQ10	Coenzyme Q10
CREB	cAMP Response Element Binding Protein
DNA	Deoxyribonucleic Acid
DON	Donepezil
DTNB	5,5-dithiobis-(2-nitrobenzoic acid)
EB	Ethidium Bromide
ECD	Electrochemical Detector
EDTA	Ethylenediaminetetraacetic Acid
ELT	Escape Latency Task
ERK	Extracellular Signal-Regulated Kinase
ETC	Electron Transport Chain
FDA	Food and Drug Administration
FNG	Fingolimod
FSK	Forskolin
HD	Huntington’s Diseases
HMG-CoA	Hydroxymethylglutaryl-Coenzyme A
HPLC	High Performance Liquid Chromatography
IACE	Institutional Animal Ethics Committee
ICP	Intracerebropeduncle
IL-1β	Interleukin-1β
i.p.	Intraperitoneal injection
LA	Locomotor Activity
LDH	Lactate Dehydrogenase
LFB	Luxol Fast Blue
MBP	Myelin Basic Protein
MDA	Malondialdehyde
MEM	Memantine
MS	Multiple Sclerosis
mtDNA	Mitochondrial DNA
MWM	Morris Water Maze
NaCl	Sodium Chloride
NAD	Nicotinamide Adenosine Dinucleotide
NADH	Nicotinamide Adenosine Dinucleotide + Hydrogen
NADPH	Nicotinamide Adenosine Dinucleotide Phosphate
NaH2PO4	Sodium Dihydrogen Phosphate
NaOH	Sodium Hydroxide
NBW	Narrow Beam Walk
NGF	Nerve Growth Factor
NMDA	N-methyl D-apartate
NMDAR	NMDA receptor
NO	Nitric Oxide
NTs	Neurotransmitters
OPA	o-phthaldehyde
OPC	Oligodendrocytes Precursor Cells
PK_A_	Protein kinase A
PMS	Post-Mitochondrial Supernatant
rpm	Revolution Per Minute
SD	Standard Deviation
SDH	Succinate Dehydrogenase
S1P	Spingosine 1-Phosphate
TNF- α	Tumor Nacrotic Factor- α
TSTQ	Time Spent in Target Quadrant
